# Randomized double-blind, placebo-controlled multicenter phase III study of prevention of irinotecan-induced diarrhea by a probiotic mixture containing Bifidobacterium BB-12^®^ *Lactobacillus rhamnosus* LGG^®^ in colorectal cancer patients

**DOI:** 10.3389/fonc.2023.1168654

**Published:** 2023-08-04

**Authors:** Michal Mego, Radoslav Danis, Jozef Chovanec, Silvia Jurisova, Branislav Bystricky, Stefan Porsok, Peter Konkolovsky, Vladimir Vaclav, Maria Wagnerova, Marian Streško, Bibiana Brezinova, Mária Rečková, Dagmar Sutekova, Natalia Pazderova, Mária Novisedlakova, Eva Zomborska, Sona Ciernikova, Daniela Svetlovska, Lubos Drgona

**Affiliations:** ^1^ Faculty of Medicine, Comenius University and National Cancer Institute, Bratislava, Slovakia; ^2^ Faculty of Medicine, Comenius University, Bratislava, Slovakia; ^3^ Department of Oncology, St. Jacob Hospital, Bardejov, Slovakia; ^4^ Department of Oncology, Faculty Hospital, Trencin, Slovakia; ^5^ Department of Oncology, Regional Cancer Center, Komarno, Slovakia; ^6^ Department of Oncology, University Hospital Milosrdni Bratia, Bratislava, Slovakia; ^7^ Department of Oncology, East Slovakia Comprehensive Cancer Center, Kosice, Slovakia; ^8^ Department of Oncology, Faculty Hospital, Trnava, Trebisov, Slovakia; ^9^ Department of Oncology, Trebisov Hospital, Trnava, Slovakia; ^10^ Department of Oncology, Regional Cancer Center, Poprad, Slovakia; ^11^ Department of Oncology, University Hospital Martin, Martin, Slovakia; ^12^ Biomedical Center, Cancer Research Institute, Slovak Academy of Sciences, Bratislava, Slovakia

**Keywords:** irinotecan, diarrhea, probiotics, colorectal cancer, beta-glucuronidase

## Abstract

**Background:**

The incidence of irinotecan-induced diarrhea varies between 60-90%, by which the incidence of severe diarrhea is 20-40%. The objective of this phase III trial was to determine the effectiveness of the probiotic mixture containing *Bifidobacterium*, BB-12^®^ and *Lactobacillus rhamnosus*, LGG^®^ in the prophylaxis of irinotecan-induced diarrhea in metastatic colorectal cancer patients due to a reduction in the activity of intestinal beta-D-glucuronidase.

**Methods:**

From March 2016 to May 2022, a total of 242 patients with colorectal cancer starting a new line of irinotecan-based therapy were registered to the study in 11 cancer centers in Slovakia. Patients were randomized in a ratio 1:1 to probiotic formula vs. placebo that was administered for 6 weeks. Each capsule of Probio-Tec^®^ BG-Vcap-6.5 contained 2.7x10^9^ colony-forming units (CFU) of 2 lyophilized probiotic strains *Bifidobacterium*, BB-12^®^ (50%) and *Lactobacillus rhamnosus* GG, LGG^®^ (50%).

**Results:**

Administration of probiotics compared to placebo was not associated with a significant reduction of grade 3/4 diarrhea (placebo arm 11.8% vs. probiotic arm 7.9%, p=0.38). Neither the overall incidence of diarrhea (46.2% vs. 41.2%, p=0.51) nor the incidence of enterocolitis (3.4% vs. 0.9%, p=0.37) was different in the placebo vs. probiotic arm. Subgroup analysis revealed that patients with colostomy had higher incidence of any diarrhea and grade 3/4 diarrhea in the placebo arm compared to the probiotic arm (48.5% vs. 22.2%, p=0.06 and 15.2% vs. 0%, p=0.06, respectively). Moreover, patients on probiotic arm had significantly better diarrhea-free survival (HR = 0.41, 95%CI 0.18 – 0.95, p=0.05) and needed less loperamide (p=0.01) compared to patients on placebo arm. We did not observe any infection caused by probiotic strains used in this study.

**Conclusion:**

This study failed to achieve its primary endpoint, and results suggest a lack of benefit of administered probiotic formula for the prevention of irinotecan-induced diarrhea. However, subgroup analysis suggests a possible benefit in patients with colostomy.

## Introduction

Diarrhea is a relatively common complication in cancer patients (1). Several mechanisms participated at its inception; malabsorption since chemotherapy-induced mucositis, dysbiosis related to the broad-spectrum antibiotics, and predisposition to infectious diarrhea in immunocompromised patients. Some anticancer chemotherapy and their metabolites can also induce diarrhea directly due to their effect on the intestinal mucosa (1, 2).

The use of probiotics in the prevention and treatment of diarrhea relies on both the theoretical assumptions and the results of several clinical trials (3). Lactic acid bacteria are involved in the treatment of dysbiosis, compete for substrate with pathogenic bacteria, produce bacteriocins, and increase transepithelial resistance (3–5). Their enzymatic activity affects the activation or deactivation of metabolites that cause diarrhea. The production of short-chain fatty acids, which are crucial for the maintenance of intestinal mucosal cells also contributes to their antidiarrheal effect (6).

Meta-analysis aimed to assess the effect of probiotics in the treatment of antibiotic-associated diarrhea (AAD) included 63 studies with a total of 8014 participants showing a significant reduction in the risk of infectious diarrhea (7). The two most studied probiotics were *Lacticaseibacillus rhamnosus* GG (previously *Lactobacillus rhamnosus* GG) (8) and *Saccharomyces boulardii*. Recent meta-analysis that included 42 trials with 11,305 participants suggests the benefit of probiotics in the prevention of AAD. The pooled analysis suggests that co-administration of probiotics with antibiotics reduces the risk of AAD in adults by 37% (9). Results of the meta-analysis of probiotics in the prevention of AAD in children showed similar benefit, while positive correlation was most prominent in studies with a higher dose of probiotics (10).

Irinotecan is a topoisomerase I inhibitor and one of the key drugs in the treatment of various malignancies, including colorectal cancer (11). The incidence of irinotecan-induced diarrhea varies between 60-90%, and the incidence of severe diarrhea is 20-40% (11). Diarrhea is a critical factor in morbidity and mortality during irinotecan-based chemotherapy (12). Predisposing factors are age over 65 years, ECOG performance status (ECOG PS) of ≥1, and previous abdominopelvic radiation (12, 13). One of the irinotecan metabolites, SN-38 (7-ethyl-10-hydroxycamptothecin), which is glucuronidated in the liver and subsequently expelled into the intestine, is the main cause of diarrhea. Bacterial beta-D-glucuronidase deconjugates SN-38 in intestinal lumen again. Deconjugated SN-38 causes direct damage to the intestinal mucosa, leading to water and electrolytes malabsorption, and the development of diarrhea (12).

Reduction of the activity of intestinal beta-D-glucuronidase using broad-spectrum antibiotics and/or beta-D-glucuronidase inhibitors represents a way to avoid irinotecan-induced diarrhea (12). It is also possible to modulate the irinotecan metabolism using cyclosporine and phenobarbital to reduce biliary excretion of SN-38 and to promote induction of glucuronidation. Promising results were shown using activated charcoal, which has resulted in the adsorption of SN-38. Furthermore, other methods were tested, including oral alkalization, thalidomide, and amifostine, but without success (12). These procedures have been studied only in phase II trials. Some probiotic bacteria reduce the activity of intestinal beta-D-glucuronidase (14, 15), and therefore these bacteria could be applied in the prevention of diarrhea in patients treated by irinotecan-based therapy.

Results of the pilot phase II study, which was prematurely terminated due to poor accrual, showed the benefit of the probiotic formula Colon Dophilus, containing 6 probiotic strains, on irinotecan-induced gastrointestinal toxicity (16). In this trial, 46 patients with colorectal cancer starting a new line of irinotecan-based therapy were enrolled. Patients were randomized to a 1:1 ratio to probiotic (PRO) or placebo arms (PLA). The probiotic formula Colon Dophilus was orally administered at a dose of 10x10^9^ CFU of bacteria three times a day for 12 weeks of chemotherapy. Administration of probiotics compared to placebo led to a statistically non-significant reduction in the incidence of severe grade 3/4 diarrhea (0% for PRO vs. 17.4% for PLA, p=0.11), as well as to a reduction in the overall diarrhea incidence (39.1% for PRO vs. 60.9% for PLA, p=0.24) and the incidence of enterocolitis (0% for PRO vs. 8.7% for PLA). Patients on PRO used fewer antidiarrheal drugs compared to PLA. There was no infection caused by probiotic strains recorded (16).

The probiotic mixture used in this trial contains two probiotic strains *Bifidobacterium*, BB-12^®^ and *Lactobacillus rhamnosus*, LGG^®^. Studies have found that the LGG^®^ strain supports immunity and decreases the incidence of gastrointestinal infections, antibiotic-associated diarrhea, and respiratory infections. In addition to its well-researched health benefits, the safety of the LGG^®^ strain has been more widely studied than any other probiotic bacterium (17).

The objective of this phase III trial was to determine the effectiveness of the probiotic formula Probio-Tec^®^ BG-Vcap-6.5 in the prophylaxis of irinotecan-induced diarrhea in metastatic colorectal cancer patients due to a reduction in the activity of intestinal beta-D-glucuronidase.

## Patients and methods

### Study patients

Eligible participants were adult patients with histologically proven colorectal cancer starting a new line of chemotherapy based on irinotecan with ECOG PS 0 – 1 at study entry. Exclusion criteria comprised impossibility to take oral medication, active infection treated by antibiotic therapy, ileostomy, hypersensitivity to study drug, and any concurrent malignancy other than non-melanoma skin cancer, no other cancer in the past 5 years. The study was registered to the clinical trials database (ClinicalTrials.gov Identifier: NCT02819960, for more details, see www.clinicaltrials.gov).

The study was approved by the Ethical committee of the National Cancer Institute, and all patients signed informed consent before enrollment in the study.

### Trial design

This was a randomized, double-blind, multicenter phase III trial to assess efficacy (as measured by prevention of grade 3/4 diarrhea during the first 6 weeks of irinotecan-based chemotherapy) of orally administered probiotic formula Probio- Tec^®^ BG-VCAP-6.5^®^ compared to placebo in patients with colorectal cancer starting a new line of irinotecan-based chemotherapy.

### Randomization and blinding

This clinical trial was conducted in 11 centers in Slovakia (National Cancer Institute in Bratislava, Stefan Kukura Hospital and Polyclinic in Michalovce, St. Jacob Hospital in Bardejov, Regional Cancer Center in Komarno, Regional Cancer Center in Poprad, Faculty Hospital in Trencin, Faculty Hospital in Trnava, University Hospital in Martin, East Slovakia Comprehensive Cancer Center in Kosice, University Hospital Milosrdni Bratia in Bratislava and Regional Hospital in Trebisov).

Patients were centrally randomized and stratified according to a treatment center. The randomization ratio was 1:1. Following the signing of informed consent, the investigator called to randomization center, and the patient received the study number. A preformed randomization table was used for patients’ allocation to one of the treatment groups (probiotic or placebo). The allocation sequence was random and concealed from investigators and patients. The investigator received the identification number of pre-supplied containers for the randomized patient, and the patient obtained corresponding containers. All containers with probiotics/placebo looked the same and were numbered sequentially in a random order using random.org. All patients, investigators, and statisticians were blinded to treatment allocation.

### Drug administration

Probiotic formula Probio-Tec^®^ BG-Vcap-6.5 (produced by Chr. Hansen A/S, Hoersholm, Denmark) was administered orally at a dose of 3x1 cps per day for 6 weeks. No premedication or patient monitoring after the administration of the probiotic formula was required. The probiotic formula might be taken after meals or snacks to reduce stomach upset. The capsule should be swallowed whole, or in case of problems with swallowing, the capsule could be opened, content mixed with a small amount of food.

Each capsule with a potency of 2.7x10^9^ CFU contained a probiotic blend of 2 lyophilized probiotic strains *Bifidobacterium*, BB-12^®^ (50%) and *Lactobacillus rhamnosus*, LGG^®^ (50%). Scientific references for these strains are *Bifidobacterium animalis* subsp. *lactis*, BB-12 (DSM 15954) and *Lacticaseibacillus rhamnosus*, LGG (DSM 33156) (8). Culture percentages are based on cell concentration and are approximate. Additives include maltodextrin, microcrystalline cellulose, silicon dioxide, magnesium salts of fatty acids, sucrose, sodium ascorbate, hypromellose, and titanium dioxide.

The placebo was manufactured by Chr. Hansen A/S and contained maltodextrin. The placebo was indistinguishable from the capsule with probiotics in terms of color, appearance, taste, smell, size, shape, and other properties and contained the same additives as a probiotic capsule. Drugs were stored at room temperature in a dry and dark place until use.

### Duration of therapy

Probiotics were administered during irinotecan-based chemotherapy for 6 weeks. Patients might also discontinue protocol therapy in the case of intercurrent illness, affecting the patient safety in the judgment of the investigator, the ability to deliver treatment or the primary study endpoints, and/or by request by the patient.

### Concomitant therapy

Patients received full supportive care during the study, including transfusion of blood and blood products, treatment with antibiotics, anti-emetics, antidiarrheal agents, analgesics, erythropoietin, or bisphosphonates, when appropriate.

### Treatment evaluation

Clinical evaluation included demography, date of birth, race, gender, medical history: cancer-specific history, including date of diagnosis, primary tumor type with histology determination, prior surgical and/or radiological therapy (date, organ/anatomic regions that have received surgical and/or radiological therapies must be documented), current stage of cancer, prior systemic therapy, ongoing toxicity related to previous treatment, history of other malignancies, other significant medical histories within past 6 months.

All toxicities, including diarrhea, enterocolitis, and all others, were evaluated utilizing NCI Common Terminology Criteria for Adverse Events Version 4.1 (CTCAE) (18). Patients filled out their diaries, which contained data related to the number and consistency of stool each day and the number and type of antidiarrheal drugs used during the study. The adherence of patients to study drugs was not evaluated.

All the patients registered in the study were accounted for follow-up. The number of patients who were not evaluable, who died or withdrew informed consent prior treatment, was specified. The distribution of follow-up time was described, and the number of patients lost to follow-up was given.

### Role of sponsor

The study sponsor (S&D Pharma) had no impact on the study design, treatment evaluation, and/or statistical analysis of the study data.

### Statistical design

#### Statistical and analytical plan

This was a phase III study to investigate the efficacy of the probiotic formula Probio-Tec^®^ BG-Vcap-6.5 given orally compared to a placebo in patients with colorectal cancer starting a new line of irinotecan-based chemotherapy.

The primary endpoint of this study was the prevention of grade 3/4 diarrhea during the first 6 weeks of irinotecan-based chemotherapy.

#### Study design, significance level, and power

H (0) –There is no difference in the absence of grade 3/4 diarrhea between the patients in the treatment and the control group (75% for both study groups)

H (A) – There is a 15% difference in the absence of grade 3/4 diarrhea between the patients in the treatment and the control group (90% for the treatment group and 75% for the control group)

Group sample sizes of 100 in group one and 100 in group two achieve 82% power to detect a difference between the group proportions of 0.1500. The proportion in group one (the treatment group) was assumed to be 0.7500 under the null hypothesis and 0.9000 under the alternative hypothesis. The proportion in group two (the control group) is 0.7500. The test statistic used was the two-sided Z test with pooled variance. The significance level of the test was targeted at 0.0500. The significance level achieved by this design was 0.0494. Because of the expected 10% of ineligibility, the proposed number of study patients was 220.

### Statistical analysis

Data were analyzed in accordance with a pre-specified plan for statistical analysis. The patients’ characteristics were summarized using the median (range) for continuous variables and frequency (percentage) for categorical variables. Kolmogorov—Smirnov test was used to assess the normality of distribution. If normally distributed, sample means were tested by Student t-test or analysis of variance (ANOVA) with Bonferroni’s or Tamhane’s corrections depending on the homogeneity of variance. The nonparametric Mann-Whitney U or Kruskal-Wallis H test was used for non-normally distributed data. Categorical data were tested by Fisher’s exact test or Chi-square test. Event-free (diarrhea) survival was calculated using Kaplan-Meier methods and compared between study arms by log-rank test. Data were calculated from day 1 of probiotic administration until the event or until the end of the study when the data were censored. All p-values presented are two-sided, and associations were considered significant if the p-value was less or equal to 0.05. Statistical analyses were performed by NCSS 2022 statistical software (Hintze J, 2022, Kaysville, UT, USA).

## Results

**Figure 1:**
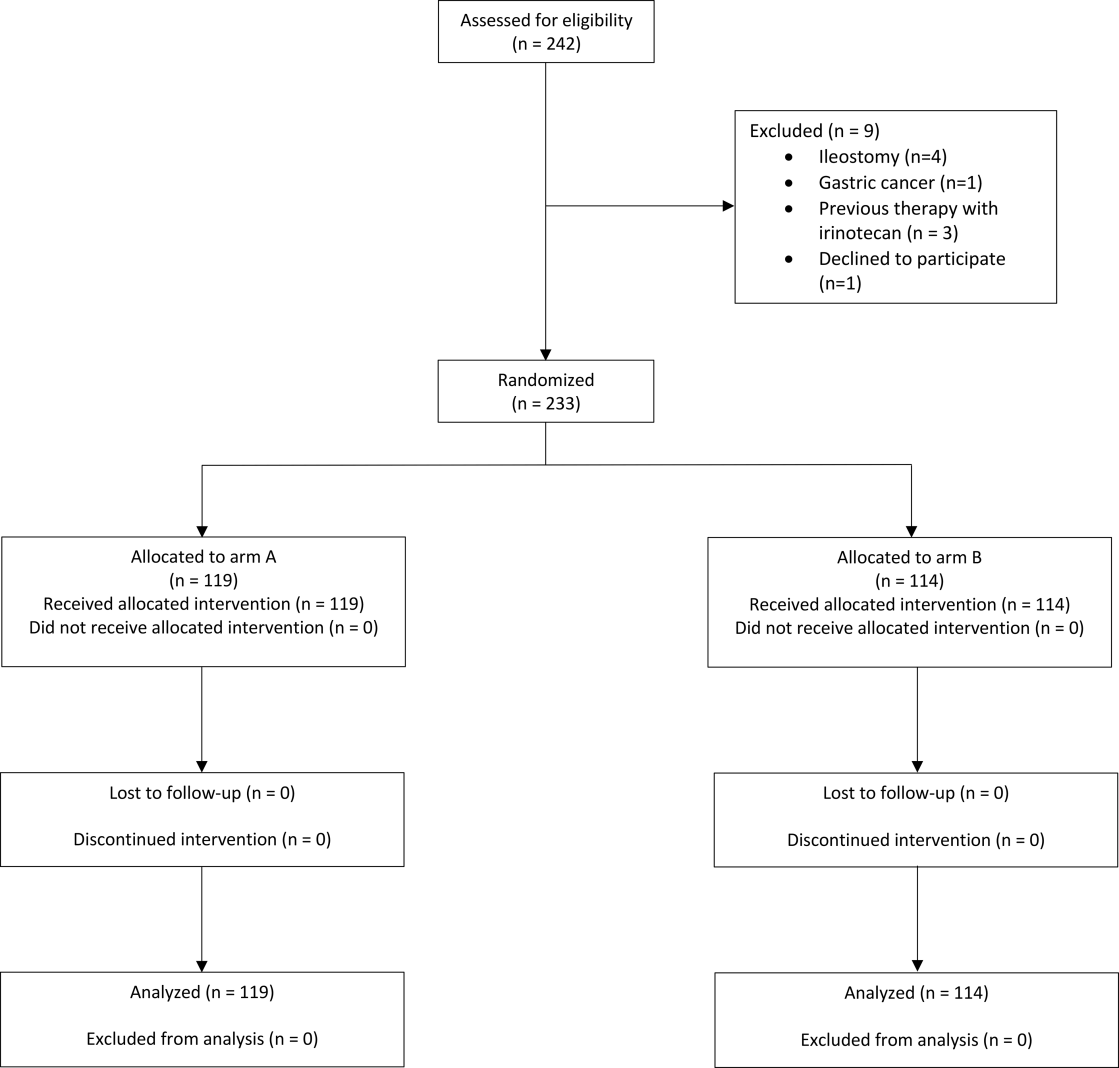
Flow diagram.

From March 2016 to May 2022, 242 patients with colorectal cancer starting a new line of irinotecan-based therapy were registered to the study in 11 cancer centers in Slovakia (**Figure 1**). Nine patients did not meet the study eligibility criteria and were excluded from intention-to-treat analysis. Reasons for ineligibility were ileostomy (4 patients), previous therapy with irinotecan (n=3), gastric cancer (1 patient), and decline to participate (1 patient). In total, 233 patients were randomized, from which 119 were allocated to the placebo and 114 to the probiotic arm, respectively.

The characteristics of patients and chemotherapy regimens are summarized in **Table 1**. There were some imbalances between treatment arms. More patients with colon cancer were allocated to the probiotic arm, opposite to rectal cancer, which was consistent with previous radiation therapy to the rectum. Slightly more patients received adjuvant therapy on the probiotic arm, while more of them were treated with first-line chemotherapy within this study. Colostomy was lightly more common in patients treated within the placebo arm. Irinotecan regimens were well-balanced between the arms, as well as 5-FU-based, anti-EGFR, and anti-VEGF therapy. Most patients received complete therapy according to a protocol (placebo arm 102 (85.7%) of patients vs. probiotic arm 103 (90.4%) of patients, p = 0.20). The reason for discontinuation of treatments was the toxicity of chemotherapy 8 (6.7%) in the placebo arm and 5 (4.4%) probiotic arm, and disease progression (placebo arm 5 [4.2%] vs. probiotic arm 1 [0.9%]).

**Table 1:** Patients’ characteristics.

Variables	Placebo		Probiotics	
	N	%	N	%
**All patients**	119	100.0	114	100.0
**Age, median (range), years**	66	(36-82)	65	(29-82)
Gender				
male	71	59.7	67	58.8
female	48	40.3	47	41.2
ECOG PS				
0	90	75.6	90	78.9
1	28	22.7	24	21.1
unkown	1	0.9	0	0.0
Tumor localization				
colon	74	62.2	80	70.2
rectum	42	35.3	33	28.9
unkown	3	2.5	1	0.9
Surgery of primary tumor				
no	24	20.2	28	24.6
yes	93	78.2	86	75.4
unkown	2	1.7	0	0.0
Colostomy				
no	86	72.3	87	76.3
yes	32	26.9	27	23.7
Previous radiotherapy to rectum				
yes	25	21.0	19	16.7
no	94	79.0	95	83.3
Previous therapy				
adjuvant chemotherapy	79	66.4	80	70.2
chemotherapy for metastatic disease	70	58.8	60	52.6
5-Fluorouracil-based including capecitabine	66	55.5	53	46.5
anti-VEGF	31	26.1	26	22.8
anti-EGFR	11	9.2	9	7.9
Current therapy				
Line of therapy				
1^st^ line	49	41.2	54	47.4
2^nd^ line	63	52.9	55	48.2
3^rd^ line	7	5.9	5	4.4
Chemotherapy				
irinotecan weekly	18	15.1	20	17.5
irinotecan every 2 weeks	85	71.4	78	68.4
irinotecan every 3 weeks	16	13.4	16	14.0
5-Fluorouracil	61	51.3	59	51.8
5-Fluorouracil bolus	14	11.8	10	8.8
5-Fluorouracil continues	45	37.8	49	43.0
Capecitabine	46	38.7	41	36.0
5-Fluorouracil-based chemotherapy	107	89.9	100	87.7
anti-VEGF	14	11.8	12	10.5
anti-EGFR	40	33.6	38	33.3

**Figure 2:**
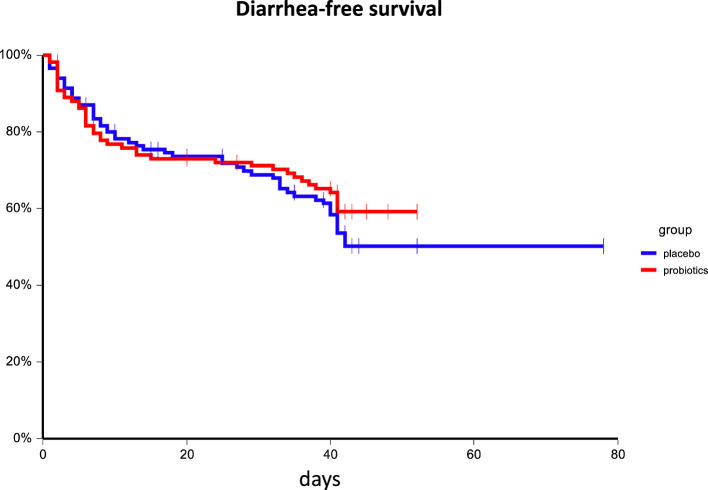
Kaplan–Meier estimates of probabilities of diarrhea-free survival according to probiotic administration (N = 233). Patients on probiotic arm had non-significantly better diarrhea-free survival as compared to patients on placebo arm HR = 0.86, 95%CI 0.57 – 1.28, p = 0.45.

**Figure 3:**
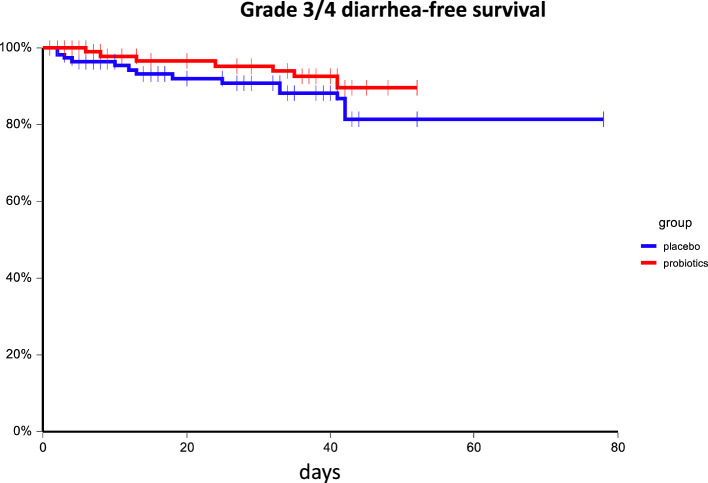
Kaplan–Meier estimates of probabilities of grade 3/4 diarrhea-free survival according to probiotic administration (N = 233). Patients on probiotic arm had non-significantly better diarrhea-free survival as compared to patients on placebo arm HR = 0.63, 95%CI 0.27 – 1.48, p = 0.30.

Administration of probiotics compared to placebo was not associated with a reduction of grade 3/4 diarrhea (placebo arm 11.8% vs. probiotic arm 7.9%, p=0.38). Similarly, the overall incidence of diarrhea did not differ between treatment arms (placebo arm 46.2% vs. probiotic arm 41.2%, p=0.51), and there was no difference in enterocolitis among groups (placebo arm: 3.4% vs. probiotic arm 0.9%, p=0.37). The incidence of bloating was similar between arms as well (**Table 2**). There was no difference in diarrhea-free survival between treatment arms (HR = 0.86, 95%CI 0.57 – 1.28, p = 0.45), and or grade 3/4 diarrhea-free survival (HR = 0.63, 95%CI 0.27 – 1.48, p = 0.30) (**Figures 2**, **3**). We received filled study diaries from 198 (85.0%) patients. There were no statistically significant differences between study arms regarding the number and consistency of stools and usage of antidiarrheal drugs (**Table 3**).

**Table 2:** Study results.

	Placebo		Probiotics		
Variables	N	%	N	%	p-value
**Diarrhea grade 3/4 (primary endpoint)**	14	11.8	9	7.9	0.38
**Diarrhea any grade**	55	46.2	47	41.2	0.51
Diarrhea (grades)					
0	64	53.8	66	57.9	0.26
1	26	21.8	17	14.9	
2	15	12.6	22	19.3	
3	13	10.9	9	7.9	
4	1	0.8	0	0.0	
**Enterocolitis**	4	3.4	1	0.9	0.37
Enterocolitis (grades)					
1	1	0.8	1	0.9	0.39
2	1	0.8	0	0.0	
3	2	1.7	0	0.0	
4	0	0.0	0	0.0	
**Abdominal bloating**	9	7.6	8	7.0	1.00

**Table 3:** Patients’ diaries.

Variables	N	mean	median	SE	SD	p-value
Number of days with normal stool						
Placebo	100	18.1	17.5	12.6	1.3	0.57
Probiotics	98	19.2	19.5	12.6	1.3	
Number of normal stools						
Placebo	100	25.9	20.5	23.4	3.0	0.76
Probiotics	98	29.5	22.0	34.8	3.0	
Number of days with mushy stool						
Placebo	100	18.7	16.5	12.8	1.2	0.37
Probiotics	98	16.8	16.0	11.8	1.2	
Number of mushy stools						
Placebo	100	37.6	28.5	40.3	3.8	0.43
Probiotics	98	33.2	23.0	34.9	3.8	
Number of days with watery stool						
Placebo	100	4.6	2.0	7.0	0.7	0.47
Probiotics	98	4.9	2.5	6.3	0.7	
Number of watery stools						
Placebo	100	14.0	3.0	36.2	3.0	0.58
Probiotics	98	13.1	3.5	23.1	3.1	
Number of days of using loperamide						
Placebo	100	3.9	0.0	10.1	0.9	0.33
Probiotics	98	2.4	0.0	6.9	0.9	
Number of loperamide tablets						
Placebo	100	9.4	0.0	26.9	2.2	0.33
Probiotics	98	4.5	0.0	15.0	2.2	
Number of days of using diphenoxylate						
Placebo	100	3.2	0.0	8.1	0.7	0.32
Probiotics	98	1.7	0.0	4.3	0.7	
Number of diphenoxylate tablets						
Placebo	100	7.2	0.0	22.0	1.7	0.31
Probiotics	98	2.8	0.0	7.6	1.7	
Number of days of using diphenoxylate and/or loperamide						
Placebo	100	7.1	0.0	14.8	1.21	0.12
Probiotics	98	4.1	0.0	8.7	1.23	
Number of diphenoxylate and/or loperamide tablets						
Placebo	100	16.5	0.0	40.7	3.15	0.11
Probiotics	98	7.4	0.0	17.8	3.18	
Diarrhea duration (days)						
Placebo	54	18.1	11.0	15.4	2.1	0.21
Probiotics	46	14.5	6.5	15.3	2.3	

**Figure 4:**
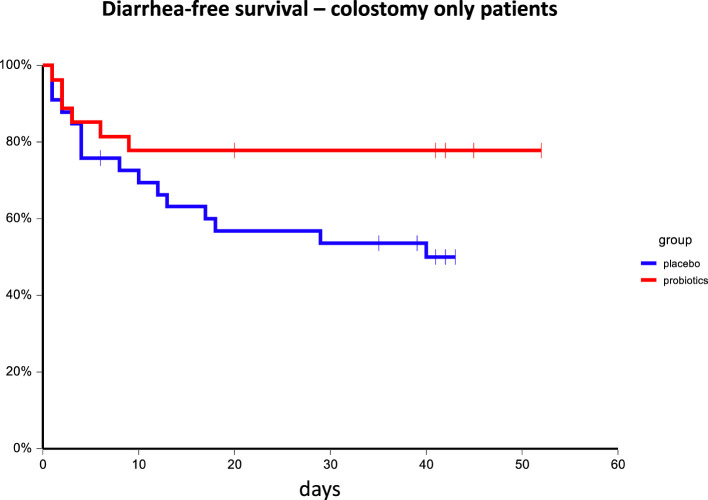
Kaplan–Meier estimates of probabilities of diarrhea-free survival in patients with colostomy according to probiotic administration (N = 233). Patients on probiotic arm had significantly better diarrhea-free survival as compared to patients on placebo arm HR = 0.41, 95%CI 0.18 – 0.95, p = 0.05.

**Figure 5:**
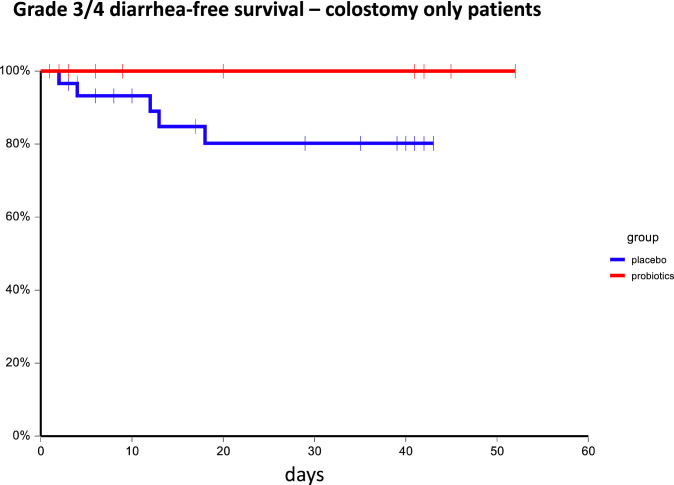
Kaplan–Meier estimates of probabilities of grade 3/4 diarrhea-free survival in patients with colostomy according to probiotic administration (N = 233). Patients on probiotic arm had significantly better diarrhea-free survival as compared to patients on placebo arm; 6-week diarrhea-free survival for probiotics vs. placebo 100.0% vs. 80.3%, p = 0.03.

In subgroup analysis, we were not able to identify any subgroup that benefits from probiotic administration except patients with colostomy. In these patients, there was a trend for the higher incidence of any diarrhea and grade 3/4 diarrhea in the placebo arm compared to the probiotic arm (48.5% vs. 22.2%, p=0.06 and 15.2% vs. 0%, p=0.06, respectively). Consistently, we observed that patients on probiotic arm had significantly better diarrhea-free survival as compared to patients on placebo arm HR = 0.41, 95%CI 0.18 – 0.95, p=0.05 as well as grade 3/4 diarrhea-free survival: 6-week diarrhea-free survival for probiotics vs. placebo 100.0% vs. 80.3%, p=0.03 (**Figures 4**, **5**). Moreover, patients with colostomy on placebo arm used more days of loperamide and received more loperamide tablets compared to patients on probiotic arm (mean ± SEM 5.74 ± 1.84 vs. 0.59 ± 2.03, p = 0.01 and 13.48 ± 4.58 vs. 1.00 ± 5.08, p = 0.01, respectively). We did not observe any infection caused by probiotic strains used in this study.

## Discussion

This large prospective double-blind placebo-controlled trial failed to achieve its primary endpoint of reduction of grade 3/4 irinotecan-based chemotherapy-associated diarrhea by probiotics. Furthermore, this trial failed to identify any subgroup with the benefit of probiotics except for the beneficial effect in patients with colostomy. These results are in contrast with a previous pilot study, suggesting a possible benefit of the probiotic formula Colon Dophilus in a similar patient population. In this study, the control group had lower than expected grade 3/4 diarrhea (11.8% vs 25.0%). Similarly, the incidence of any diarrhea (43.8%) was lower compared to the previous study as well as to the literature (13). This could be attributable to a slightly different patient population, an especially lower proportion of patients with colostomy in this trial compared to the previous one. Moreover, substantially more patients received a weekly irinotecan regimen, which was associated with a higher incidence of diarrhea, in the previous study compared to the current study (60.9% vs. 16.3%). On the contrary, in the presented study, more patients received anti-EGFR inhibitors and capecitabine, and fewer anti-VEGF inhibitors compared to the pilot study (16). However, other factors, including imbalances of known risk factors (e.g., primary tumor location, presence of colostomy, pretreatment with adjuvant chemotherapy and/or radiation therapy) and unknown confounding factors could affect study results as well. Another factor that could be responsible for this difference is the type and the potency of utilized probiotic formula. Furthermore, other mechanisms of diarrhea not related to beta-glucuronidase activity could be responsible for the lack of treatment effect as well. Consistently, with the previous trial, we didn’t observe clinically relevant toxicity related to probiotic administration.

Results of clinical trials aimed to determine the effect of probiotics in the prevention and treatment of chemotherapy-associated diarrhea are inconsistent. The results of a meta-analysis that included 7 trials with 1091 participants showed no effectivity of probiotics in the prevention of any diarrhea or severe diarrhea caused by anticancer treatment, including chemotherapy, targeted therapy, and immunotherapy compared to placebo. In addition, administration of probiotics did not influence the usage of salvage antidiarrheal medication (19). Similar results produced a meta-analysis based on randomized trials rated as having a low risk of bias (Danis et al., 2022). On the contrary, another meta-analysis suggests the beneficial effect of probiotics on diarrhea in cancer patients (20). This meta-analysis included 2982 adult and pediatric cancer patients. According to the findings, probiotics may reduce the incidence of diarrhea in patients with cancer (odds ratio OR = 0.52, 95% CI 0.34-0.78), but further studies are needed (20). Moreover, safety analysis demonstrated only 5 cases with bacteremia/fungemia/positive blood culture associated with the probiotic intervention (20).

The microbiota-host-irinotecan axis describes the interaction between the gut microbiome, host immune microenvironment, and host drug metabolism after irinotecan chemotherapy (21). Animal models focusing on irinotecan administration documented changes in microbiota composition after the treatment. This included the increased presence of intestinal *Enterobacteriaceae* spp. and *Clostridium* cluster XL (22) accompanied by an increase of pro-inflammatory cytokines and changes in mucosa composition with reduced adhesion sites. These changes lead to a reduction in the number of symbiotic bacteria and an increase in the number of opportunistic pathogens (23). Numerous preclinical data suggest the beneficial effect of probiotics on irinotecan-induced gastrointestinal toxicity (6, 21, 24–29). However, clinical evidence of the beneficial effect of probiotics in this setting is limited. A prospective observational trial suggests that *Lentilactobacillus kefiri* LKF01 (Fefibios^®^) can ameliorate irinotecan-induced severe diarrhea in cancer patients (30). In contrast, a phase II/III, randomized, double-blind, placebo-controlled study failed to achieve its primary endpoint of reduction of grade 3/4 irinotecan-induced diarrhea utilizing a high-concentration multi-strain probiotic supplement (31). This observation is consistent with our study, even though the pilot study with different probiotic formulas indicated potential benefit (16).

This study has several strengths as well as some limitations. First, this is a large randomized, double-blind, multicenter study, adequately powered to answer the research question. Furthermore, patients’ treatment reflects current clinical practice in the community of oncology providers and was not restricted to academic centers. The chosen probiotic formula has the advantage to be stored at room temperature eliminating the confounder effect of the storage conditions on study results. Limitations include a lack of treatment adherence measurement as well as a lack of evaluation of gut colonization by probiotic formula and microbial composition in control group and/or measurement of stool beta-glucuronidase activity, the possible surrogate endpoints of probiotic efficacy. Further research should include mechanistic studies aimed to prove an association between stool beta-glucuronidase activity and the risk of irinotecan-induced diarrhea, as these are still lacking, as well as evaluation of different probiotic formulas and/or fecal microbiome transfer aimed to decrease the incidence of irinotecan chemotherapy-associated diarrhea.

## Conclusions

In conclusion, this large, multicenter, double-blind phase III study failed to achieve its primary endpoint, and study results suggest a lack of benefit of the probiotic mixture containing *Bifidobacterium*, BB-12^®^ and *Lactobacillus rhamnosus*, LGG^®^ on prevention of irinotecan-induced diarrhea. Subgroup analysis suggests possible benefit in patients with colostomy. These results can’t exclude the potential beneficial effect of gut microbiome modification by other probiotic formulas and/or fecal microbiota transplantation in the study patient population treated with irinotecan-based chemotherapy. Despite the very low incidence of irinotecan-induced severe diarrhea observed in our study, an effective preventive measure of this condition remains an unmet clinical need. Preservation of healthy microbiota composition could be the simple, effective, and nontoxic approach to reducing gastrointestinal toxicity of irinotecan-based chemotherapy.

## Data availability statement

The original contributions presented in the study are included in the article/supplementary material. Further inquiries can be directed to the corresponding author.

## Ethics statement

The studies involving human participants were reviewed and approved by Ethical committee of the National Cancer Institute. The patients/participants provided their written informed consent to participate in this study.

## Author contributions

MM and LD participated in the conception and design of this study. RD, SC and DSv participated in data validation. JC, SJ, BrB, SP, PK, VV, MW, MS, BiB, MR, DSu, NP, MN, EZ acquired, analyzed, and interpreted the data. MM drafted the article. All listed authors participated in critical discussions. All authors contributed to the article and approved the submitted version.
